# Geographic determinants of colorectal cancer in Louisiana

**DOI:** 10.1007/s10552-021-01546-7

**Published:** 2022-01-07

**Authors:** Denise Danos, Claudia Leonardi, Xiao-Cheng Wu

**Affiliations:** School of Public Health, Louisiana State University Health Sciences Center, New Orleans, LA USA

**Keywords:** Colorectal cancer, Rural, Socioeconomic status, Geographic disparities

## Abstract

**Purpose:**

Currently, rural residents in the United States (US) experience a greater cancer burden for tobacco-related cancers and cancers that can be prevented by screening. We aim to characterize geographic determinants of colorectal cancer (CRC) incidence in Louisiana due to rural residence and other known geographic risk factors, area socioeconomic status (SES), and cultural region (Acadian or French-speaking).

**Methods:**

Primary colorectal cancer diagnosed among adults 30 years and older in 2008–2017 were obtained from the Louisiana Tumor Registry. Population and social and economic data were obtained from US Census American Community Survey. Rural areas were defined using US Department of Agriculture 2010 rural–urban commuting area codes. Estimates of relative risk (RR) were obtained from multilevel binomial regression models of incidence.

**Results:**

The study population was 16.1% rural, 18.4% low SES, and 17.9% Acadian. Risk of CRC was greater among rural white residents (RR Women: 1.09(1.02–1.16), RR Men: 1.11(1.04–1.18)). Low SES was associated with increased CRC for all demographic groups, with excess risk ranging from 8% in Black men (RR: 1.08(1.01–1.16)) to 16% in white men (RR: 1.16(1.08–1.24)). Increased risk in the Acadian region was greatest for Black men (RR: 1.21(1.10–1.33)) and women (RR: 1.21(1.09–1.33)). Rural–urban disparities in CRC were no longer significant after controlling for SES and Acadian region.

**Conclusion:**

SES remains a significant determinant of CRC disparities in Louisiana and may contribute to observed rural–urban disparities in the state. While the intersectionality of CRC risk factors is complex, we have confirmed a robust regional disparity for the Acadian region of Louisiana.

**Supplementary Information:**

The online version contains supplementary material available at 10.1007/s10552-021-01546-7.

## Introduction

Geographic disparities in cancer incidence and outcomes can be due to numerous factors. At the individual level, demographic factors, occupation, poverty, and health behaviors or beliefs can contribute to cancer risk [1–3]. Additionally, there are many well-established cancer risk factors beyond the individual level, including healthcare access, living environment (social and physical), and large-scale policy and systems [2]. Currently, in the United States (US) residents in rural or non-metropolitan areas experience greater cancer burden for tobacco-related cancers and cancers that can be prevented by screening [4]. While national trends show the rural–urban disparity in colorectal cancer (CRC) incidence has narrowed greatly over several decades, disparities remain [4–7]. However, there is variation in geographic disparities, which underscores the need for cancer reporting at the regional level [8].

In the US, the risk of colorectal cancer has also been associated with low socioeconomic status (SES), both at the individual and area level [9–11]. Reasons for this association are complex and include higher prevalence of modifiable CRC risk factors among individuals of low SES, such as poor diet, low physical activity, and tobacco use [12, 13]. SES gradients in CRC are also influenced by healthcare access and setting, insurance status, and the ability to attend routine or follow-up medical appointments [9]. Further evidence supports spatial clustering of CRC incidence and mortality in areas of high poverty and thus may play an important role in geographic disparities in CRC [12, 14, 15].

The National Cancer Institute (NCI) has now validated small area-based measures of rural residence and SES for cancer reporting in the Surveillance, Epidemiology, and End Results (SEER) program [16, 17]. Previously, these measures were reported at the county level which has been shown to be vulnerable to aggregation and misclassification bias [18]. Importantly, small area-based measures of exposures enable the use of multilevel or hierarchical modeling, which accounts for interdependence of individuals in shared environments or systems and is well suited but underutilized in the study of rural cancer disparities [2, 18, 19]. A national assessment of CRC risk for rural residential status using small area-based measures (census tract) has not yet been reported. While a census tract-level analysis of cancer incidence found higher SES was associated with increased breast and prostate cancer incidence and lower SES was associated with increased lung cancer risk, there was no clear association between SES and colorectal cancer incidence at the national level [16].

Louisiana ranks 4th in the US for CRC incidence, with significantly greater rates of CRC among all race and sex groups when compared to national rates. Recent research has identified two distinct geographically determined risk factors for CRC in Louisiana. First, the Acadian region of south Louisiana has been shown to have experienced significantly higher rates of CRC incidence compared to state and national rates, which have motivated theories of genetic risk among the Acadian founder population [20]. Acadian settlers arrived in Louisiana in the late-18th century after being exiled from present day Nova Scotia [21]. Acadians are a subset of Louisiana Creole which is a broad term that refers to the blend of ancestry and culture (European, West African, and Native American) in the state during this period [22]. During Segregation and the Jim Crow era, white Acadian Creoles began to distance themselves from the broader mixed-race Louisiana Creole label by identifying only as Acadian or ‘Cajun’ [22]. In the 1920’s, in a push for Americanization, Louisiana school children began to be punished for speaking French at school which marked a decline in the use of Cajun and Creole dialects [23]. An ecological analysis of CRC rates during 2005–2009 found that Louisiana counties in which at least 10% of the population were French or Cajun French-speaking (excluding French Creole) had greater risk than the state average [20]. This risk was most pronounced for white men, where the incidence of CRC was 37% greater than national rates [20]. Additionally, previous research also reported a significant association between neighborhood-concentrated disadvantage, an index of socioeconomic disadvantage, and the incidence of CRC in the state [11]. In this study, we aim to characterize multiple geographic determinants of colorectal cancer incidence in Louisiana. We will examine risk differences by residential location (urban/rural), area socioeconomic status, and cultural region using multilevel analysis. Continued monitoring of geographic CRC disparities will provide insight into how the state compares to national trends and support longitudinal reporting for CRC awareness and prevention in Louisiana.

## Methods

### Data

Data on primary colorectal cancer diagnosed in Louisiana residents, 30 years and older, between 1 January 2008 and 31 December 31 2017 were obtained from the Louisiana Tumor Registry, a participant of the NCI’s SEER Program and the Centers for Disease Control and Prevention’s National Program of Cancer Registries. Cases were identified by the International Classification of Diseases for Oncology, Third Edition (ICD-O-3) site codes C180-C189, C199, and C209. Histology codes (9050-9055, 9140, 9590-9992) were excluded. Age was categorized into 5-year groups beginning at 30 years old. Early onset colorectal cancer (EOCRC) included cases diagnosed before 50 years old, while average-onset colorectal cancer (AOCRC) included cases in residents 50 and older. Patients were geocoded to 2010 US Census tracts by address at the time of diagnosis. Population at risk for each age, race, and sex group was determined by census tract using US Census American Community Survey (ACS) 2012 5-year population estimates. Geographic determinants were linked to patients by 2010 US Census tract. Census tracts with missing geographic data were excluded (*n* = 52). This research was approved by Louisiana State University Health Sciences Center, New Orleans Institutional Review Board.

### Geographic Determinants

**Fig. 1:**
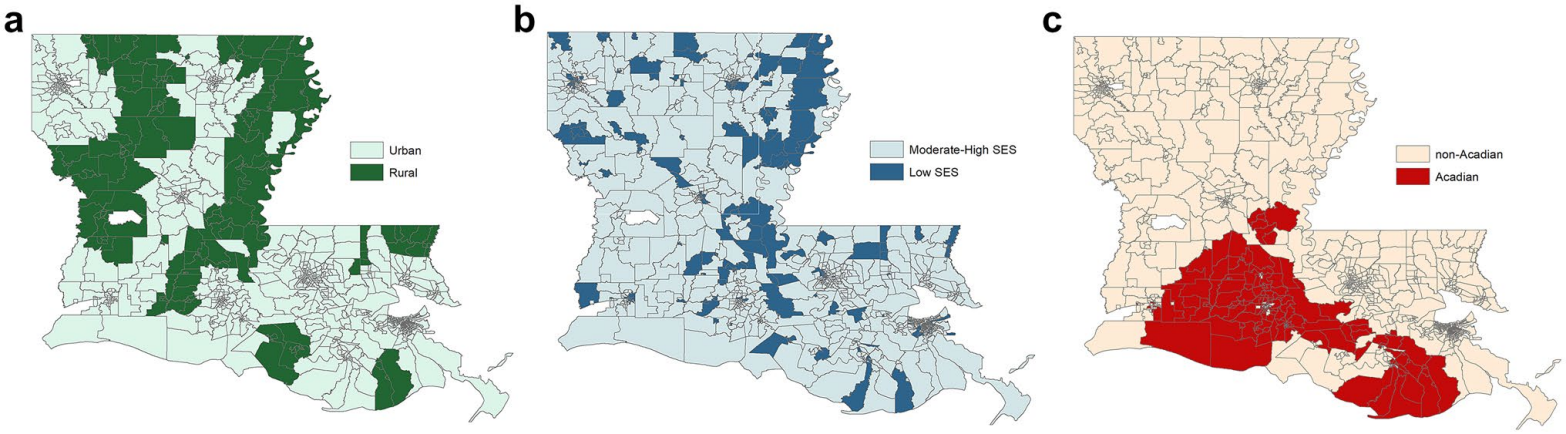
Census tract maps of geographic risk factors in Louisiana, 2008–2017; **a** rural residence, based on US Department of Agriculture 2010 rural–urban commuting area codes, **b** low socioeconomic status, based on US Census American Community Survey 2012 5-year estimates, and **c** Acadian or French-speaking areas, based on US Census American Community Survey 2012 5-year estimates

All geographic determinants were observed at the census tract level. An indicator of rural residence was derived from US Department of Agriculture 2010 rural–urban commuting area (RUCA) codes (2019 revision), with metropolitan cores and associated commuting areas (secondary codes 1.0, 1.1, 2.0, 2.1, 3.0, 4.1, 5.1, 7.1, 8.1, 10.1) classified as urban and all other areas classified as rural (Fig. 1a). While this definition of rural is consistent with the NCI’s SEER working group census tract-level study [17], a sensitivity analysis was designed to investigate the effect of metropolitan status to facilitate a comparison between our results and studies that use county-level US Department of Agriculture Rural Urban Continuum codes [4, 6–8]. For the sensitivity analysis, census tracts within metropolitan cores were identified by RUCA primary code 1 and all other census tracts were classified as non-metropolitan. An index of socioeconomic status was developed using US Census ACS 2012 5-year estimates and includes measures of occupation, unemployment, poverty, income, education, and home and rent values [24]. This index was validated by NCI and described as having a more consistent interpretation across geographic regions when compared to a competing composite index [16]. Low SES was defined as census tracts in the lowest quartile of the SES index (Fig. 1b). The Acadian region of Louisiana was defined as census tracts with more than 5% of households speaking French or Cajun French at home (Fig. 1c), based on the US Census ACS 2012 5-year estimates [20].

### Statistical Analysis

CRC incidence was analyzed as a rate of cases out of the total person-years at risk and modeled using multilevel binomial regression, with individuals nested within census tracts. Age was included in the model as a covariate and a random intercept for census tracts was used to account for correlation among residents living in the same tract. Models were stratified by race and sex. Races other than white and Black or African American were excluded due to insufficient numbers. We provide age-adjusted risk estimates for each geographic risk factor separately (rural residence, low socioeconomic status, Acadian) and then provide risk estimates conditioned on all three factors. Multilevel models were executed using GLIMMIX procedure in SAS version 9.4.

## Results

The study included 23,345 incident cases of CRC among residents in 1,096 census tracts. Characteristics of the at-risk study population and cases are presented as Table 1. Overall, the study population was 47.5% male and 29.8% Black or African American, while cases of CRC were 53.5% male and 32.4% Black or African American. Regarding geographic risk factors, 16.1% resided in rural census tracts and 18.4% resided in low-SES areas, with 6.1% of residents were in rural, low-SES tracts. Many of the rural, low-SES areas were located in the Mississippi Delta region of the state (Fig. 1a and b). 17.9% of the study population resided in Acadian or French-speaking areas (Fig. 1c).

**Table 1:** Characteristics of colorectal cases and population controls included in the study, Louisiana 2008–2017

	All					30–49					50 and over				
	Cases		Population controls		*p* value	Cases		Population controls		*p* value	Cases		Population controls		*p* value
	%	*n*	%	*n*		%	*n*	%	*n*		%	*n*	%	*n*	
All	100.0	23,345	100.0	2,442,594		100.0	2,336	100.0	1,101,165		100.0	21,009	100.0	1,341,429	
Sex					** < .0001**					0.9036					** < .0001**
Female	46.5	10,845	52.6	1,284,036		50.6	1,183	50.8	559,034		46.0	9,662	54.0	725,002	
Male	53.5	12,500	47.4	1,158,558		49.4	1,153	49.2	542,131		54.0	11,347	46.0	616,427	
Race					** < .0001**					**0.0018**					** < .0001**
White	67.6	15,773	70.2	1,714,398		63.9	1,493	66.9	737,210		68.0	14,280	72.8	977,188	
Black	32.4	7,572	29.8	728,196		36.1	843	33.1	363,955		32.0	6,729	27.2	364,241	
Rural					** < .0001**					0.0531					** < .0001**
No	82.0	19,149	83.9	2,050,134		83.2	1,943	84.6	931,821		81.9	17,206	83.4	1,118,313	
Yes	18.0	4,196	16.1	392,460		16.8	393	15.4	169,344		18.1	3,803	16.6	223,116	
Low SES					** < .0001**					** < .0001**					** < .0001**
No	77.4	18,073	81.6	1,994,131		79.0	1,845	82.2	905,048		77.2	16,228	81.2	1,089,083	
Yes	22.6	5,272	18.4	448,463		21.0	491	17.8	196,117		22.8	4,781	18.8	252,346	
Acadian					** < .0001**					**0.0052**					** < .0001**
No	80.1	18,703	82.1	2,006,182		79.7	1,862	81.9	902,245		80.2	16,841	82.3	1,103,937	
Yes	19.9	4,642	17.9	436,412		20.3	474	18.1	198,920		19.8	4,168	17.7	237,492	

*SES* socioeconomic status

Statistically significant results, at alpha = 0.05, are shown in bold

The study population included individuals 30 years or older, 55.3% of which were 50 or older. The majority of CRC cases in the study (90%, *n* = 21,009) were diagnosed in patients 50 or older and were considered average-onset colorectal cancer (AOCRC). The remaining 10% of cases were early-onset colorectal cancer (EOCRC).

**Fig. 2:**
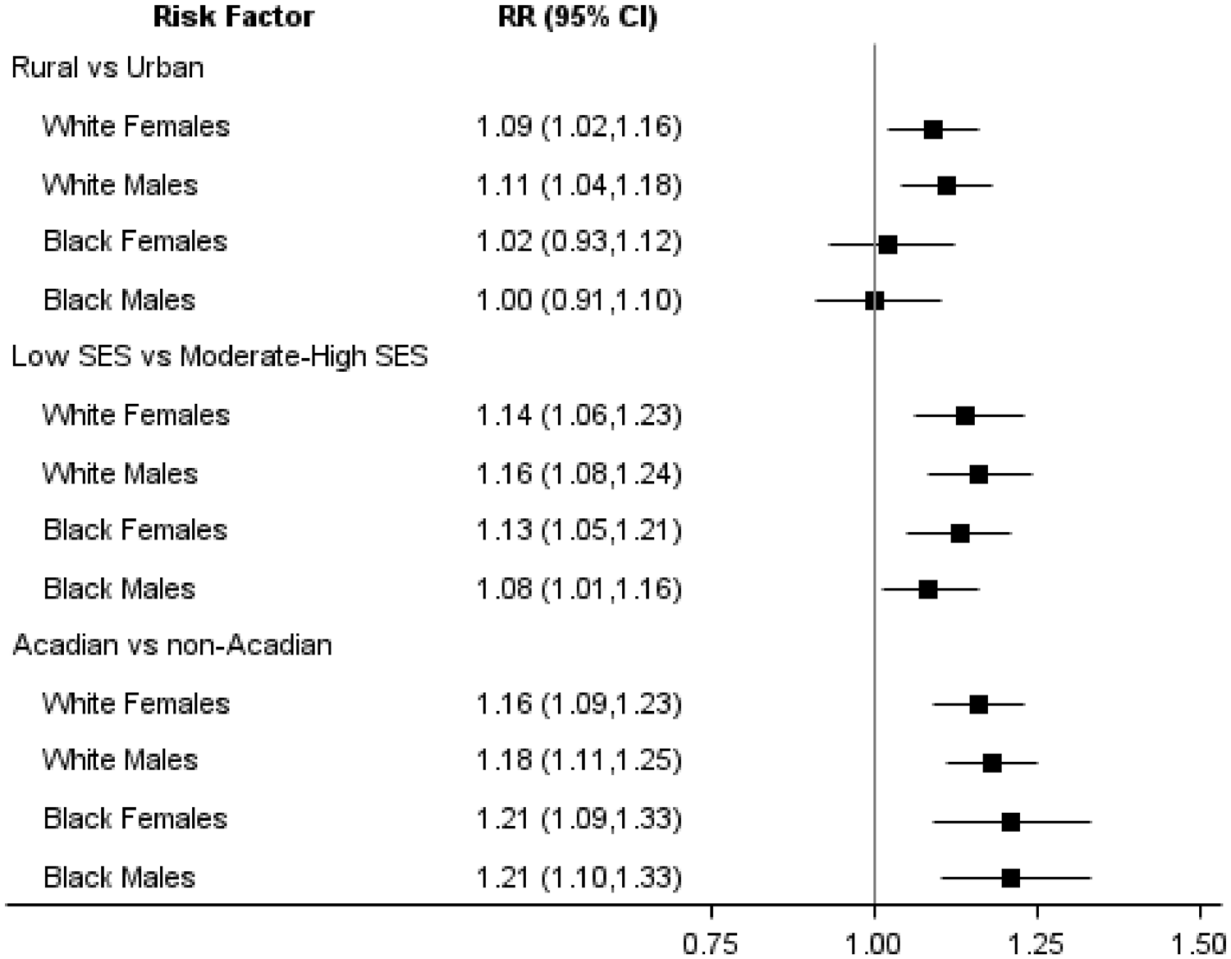
Age-adjusted relative risk (RR) estimates and 95% confidence intervals from multilevel models of colorectal cancer incidence, Louisiana 2008–2017

Estimates of risk from multilevel models of incidence are provided in Table 2. Age-adjusted risk estimates are provided in Fig. 2. For all CRC, rural–urban disparities were observed in white women and men, where the relative risk (RR) and 95% confidence interval for rural areas compared to urban was 1.09 (1.02–1.16) and 1.11 (1.04–1.18), respectively. There were no significant rural–urban disparities in Black or African American women or men. Low SES was associated with increased CRC in all race and sex groups, with the excess risk ranging from 8% in Black men [RR: 1.08 (1.01–1.16)] to 16% in white men [RR: 1.16 (1.08–1.24)] when compared to residents of moderate-to-high-SES areas. The increase in risk observed for the Acadian or French-speaking region was greatest for Black or African American men [RR: 1.21 (1.10–1.33)] and women [RR: 1.21 (1.09–1.33)]. There was also significant regional risk among white men [RR: 1.18 (1.11–1.25)] and white women [RR: 1.16 (1.09–1.23)]. Conditioned on other risk factors, rural–urban disparities in CRC among whites were no longer statistically significant [Women RR: 1.05 (0.98–1.12), Men RR: 1.06 (1.00–1.13)]. However, risk associated with low SES remained significant with risk ratios ranging from 1.10 (1.02–1.18) in Black men to 1.14 (1.07–1.23) in Black women. Similarly, Acadian or French-speaking areas had significantly increased risk, with risk ratios ranging from 1.15 (1.08–1.22) in white women to 1.22 (1.11–1.35) in Black women and men.

**Table 2:** Relative risk estimates and 95% confidence intervals from multilevel models of early and average-onset colorectal cancer incidence, Louisiana 2008–2017

	All colorectal cancer (Aged 30 and older)		Early onset colorectal cancer (Aged 30–49)		Average-onset colorectal cancer (Aged 50 and older)	
	RR (95% CI)^a^	RR (95% CI)^b^	RR (95% CI)^a^	RR (95% CI)^b^	RR (95% CI)^a^	RR (95% CI)^b^
White females						
Rural	**1.09 (1.02,1.16)**	1.05 (0.98,1.12)	1.15 (0.93,1.43)	1.04 (0.83,1.29)	**1.08 (1.01,1.16)**	1.05 (0.98,1.13)
Low SES	**1.14 (1.06,1.23)**	**1.11 (1.03,1.20)**	**1.45 (1.14,1.84)**	**1.40 (1.09,1.80)**	**1.12 (1.03,1.21)**	**1.09 (1.00,1.18)**
Acadian	**1.16 (1.09,1.23)**	**1.15 (1.08,1.22)**	**1.29 (1.07,1.56)**	**1.26 (1.05,1.52)**	**1.14 (1.07,1.22)**	**1.14 (1.06,1.21)**
White males						
Rural	**1.11 (1.04,1.18)**	1.06 (1.00,1.13)	1.18 (0.96,1.44)	1.13 (0.92,1.40)	**1.10 (1.03,1.18)**	1.05 (0.99,1.13)
Low SES	**1.16 (1.08,1.24)**	**1.12 (1.04,1.21)**	1.21 (0.95,1.53)	1.15 (0.90,1.48)	**1.15 (1.07,1.24)**	**1.12 (1.04,1.21)**
Acadian	**1.18 (1.11,1.25)**	**1.17 (1.10,1.24)**	1.07 (0.88,1.29)	1.05 (0.87,1.27)	**1.19 (1.12,1.27)**	**1.18 (1.11,1.26)**
Black females						
Rural	1.02 (0.93,1.12)	0.98 (0.89,1.07)	1.07 (0.83,1.39)	1.06 (0.81,1.37)	1.02 (0.92,1.12)	0.96 (0.87,1.07)
Low SES	**1.13 (1.05,1.21)**	**1.14 (1.07,1.23)**	1.09 (0.90,1.32)	1.08 (0.89,1.31)	**1.14 (1.05,1.22)**	**1.16 (1.07,1.25)**
Acadian	**1.21 (1.09,1.33)**	**1.22 (1.11,1.35)**	0.94 (0.71,1.24)	0.94 (0.71,1.25)	**1.25 (1.12,1.38)**	**1.27 (1.14,1.41)**
Black males						
Rural	1.00 (0.91,1.10)	0.97 (0.88,1.06)	0.95 (0.70,1.29)	0.92 (0.67,1.26)	1.01 (0.91,1.11)	0.97 (0.88,1.07)
Low SES	**1.08 (1.01,1.16)**	**1.10 (1.02,1.18)**	1.02 (0.81,1.29)	1.05 (0.83,1.33)	**1.09 (1.01,1.17)**	**1.10 (1.02,1.19)**
Acadian	**1.21 (1.10,1.33)**	**1.22 (1.11,1.35)**	1.31 (0.97,1.77)	1.32 (0.98,1.79)	**1.20 (1.08,1.32)**	**1.21 (1.10,1.34)**

*RR* relative risk, *CI* confidence interval, *SES* socioeconomic status

^a^Adjusted for age (5 year groups)

^b^Adjusted for age (5 year groups) and other risk factors in the table (rural, low SES, Acadian)

Estimates that were statistically significant, at alpha = 0.05, are shown in bold

Risk patterns in overall CRC largely reflect risk observed for AOCRC (Table 2). Conditioned on other geographic risk factors, there was increased AOCRC in both low SES and Acadian or French-speaking areas for all four major race and sex groups. Risk factors for EOCRC were identified among white women only, where rates in low-SES areas were 45% greater than those in moderate- and high-SES areas [RR: 1.45 (1.14–1.84)] and the Acadian region exhibited 29% greater rates than other areas [RR: 1.29 (1.07–1.56)]. Conditioned on other geographic risk factors, these effects remained significant.

Results from sensitivity analyses with metropolitan status are provided as supplemental Table 1. Similar to the urban–rural classification, white residents in non-metropolitan areas had greater CRC risk compared to their counterparts in metropolitan areas [RR women: 1.13 (1.08–1.20), RR men: 1.16 (1.10–1.22)]. In contrast to the urban–rural classification, disparities by metropolitan status persisted among white residents after adjusting for low SES and Acadian region [RR women: 1.09 (1.03–1.15), RR men: 1.11 (1.06–1.17)]. There were no significant disparities by metropolitan status in Black or African American residents [RR women: 1.03 (0.96–1.11), RR men: 1.03 (0.96–1.11)].

## Discussion

The present study identified an increased risk of CRC among rural white residents in Louisiana, who had 8–10% greater risk of average-onset CRC than their urban counterparts. Sensitivity analysis comparing only metropolitan cores to other geographic areas revealed a greater disparity, with between 13 and 17% increased risk observed among white residents in non-metropolitan areas. This was very consistent with national estimates for this racial group, which range from 12 to 17% [7]. We did not observe an association between rural residence or metropolitan status and CRC in Black or African American residents. This is in contrast to national estimates, where excess average-onset CRC risk in rural areas ranged from 16 to 23% in Blacks [7]. National time trend analysis suggests rural–urban disparities in CRC among Black residents are the result of greater reduction in incidence in metropolitan areas in recent years starting in 2008, a phenomenon that likely varies in temporality and magnitude across the US [4, 7]. A recent study of early-onset CRC reported extremely high rates among Black residents in rural Georgia, which were more than double rates in Black residents of California [8]. The current study found no evidence of rural–urban disparities in CRC among Black residents of Louisiana during 2008–2017, which supports the conclusion that intra-racial disparities are complex and may be time-dependent. Conclusions in this study may also be limited due to the relatively small number of Black residents living in rural areas of Louisiana (15.7%, *n* = 115,290). Thus, a longitudinal study would be useful in characterizing intra-racial geographic disparities in this population.

We found a significant increase in CRC risk among residents of low-SES areas, across all four major race and sex groups in the study, which is consistent with other studies of CRC incidence in the US [5, 10, 25]. Rural census tracts in the study were disproportionately low SES when compared to urban census tracts (42.4% vs 21.8%), and effects of low SES did appear to mediate rural–urban differences seen among the white population. Due to the importance of screening in CRC prevention, trends in incidence can reflect trends in healthcare access, which also correlate with individual SES measures, such as education, income, and insurance status [26, 27]. While rural and low-SES populations both exhibit increased prevalence of behavioral risk factors like tobacco use, diet/obesity, and physical activity [28, 29], a recent study of medical expenditures reported that unmet medical needs were more likely to differ by SES rather than rurality [30].

The ability to identify significant geographic determinants for EOCRC was limited by relatively low incidence and thus less model precision when compared to AOCRC. Age-adjusted EOCRC incidence during the study period was 20.5 per 100,000 compared to 149.3 per 100,000 for AOCRC. However, we did report significant EOCRC risk among low-SES white females, who had 40–45% increased risk compared to their moderate- and high-SES counterparts. In the US, rates of EOCRC have been greatest in Southern states and among African Americans [8, 31]. While it is important to note that the absolute incidence of EOCRC is still low, evidence of significant birth cohort effects, and changes in behavioral risk factors such as diet, metabolic dysfunction, heavy alcohol consumption, and smoking indicate a need for continued research regarding the incidence of EOCRC [32, 33].

Results from this study also confirmed significantly high rates of CRC in the Acadian region of Louisiana. This region had 16–18% increase in CRC risk among white residents and 21% increase in risk among Black or African American residents, when compared to the rest of the state. This is in contrast to previous reports of elevated CRC risk in the region among white males only [20]. Differences in methodology of the present study include a census tract rather than county designation for Acadian communities, extended study period, and multilevel analysis of risk which is well suited for assessing geographic risk because it accounts for interdependence of individuals with shared environmental context [2, 18, 19]. Defining the Acadian region based on the proportion of French-speaking households was previously intended as a proxy for Cajun ancestry to investigate the hypothesis of hereditary CRC in the Cajun population [20]. However, with significant regional risk seen across all major demographic groups, it may be this definition serving as a proxy for other cultural or broad environmental factors in the area. The Acadian region did have a greater proportion of rural census tracts when compared to the rest of the state (23.8% vs 14.8%), but the regional disparity was robust after conditioning on rurality and SES.

### Limitations

One limitation to the study is that there is no universal definition for rural in the US. In county-level studies, researchers often employed a metropolitan and non-metropolitan classification based on USDA Rural Urban Continuum codes and more nuanced categories of rural, urban or suburban, and metropolitan have been effective in characterizing geographic health disparities [4, 6–8, 18]. The urban and rural classification we used in the study were chosen to be consistent with SEER working group to support continuity in NCI cancer registry research [17]. Further, the study was limited to a 10-year period in a single state with a moderate rural population and thus lacked statistical power to sufficiently investigate the intersection of rurality, socioeconomic status, and region. Sensitivity analyses with effect interactions did not result in significant interactions and were not conclusive. While the use of cancer registry and other population-representative data sources enabled a comprehensive assessment of the population, the study concept did not include individual-level social and behavioral risk factors which may provide insight regarding mechanisms of geographic risk. Finally, the study did not include Hispanics, American Indian/Alaska Native, and Asian/pacific islanders as a subgroup for analysis due to relatively low numbers of residents in Louisiana.

## Conclusion

SES remains a significant determinant of disparities in CRC incidence in Louisiana and may contribute to observed rural–urban disparities in the state. Results from this study support efforts for prevention and control that consider how these factors interact. For example, the Louisiana Colorectal Cancer Round Table, a coalition for colorectal cancer prevention and awareness, has conducted studies to better identify differential healthcare access in the state. One study estimated that over half of GI providers in Louisiana did not accept Medicaid in 2017, the time of the State Medicaid expansion, and that the geographic distribution of providers likely affected differential rates of screening and incidence for low income and rural populations [34]. Other research has also suggested that factors regarding patient volume or payer policies matter more than location alone [27, 35, 36]. Factors other than screening can also contribute to SES and regional disparities, such as diet, physical activity, tobacco and alcohol use, or environmental exposures [9]. While the intersectionality of CRC risk factors is complex we have confirmed a regional disparity for the Acadian or French-speaking region of Louisiana for all major demographic groups in the state.

## Supplementary Information

Below is the link to the electronic supplementary material.

Supplementary file1 (DOCX 14 kb)

## Data Availability

Data are maintained by Louisiana Tumor Registry and is not publicly available. Use of the data for research must be approved by the Louisiana Tumor Registry Board of Research.

## Code availability

SAS code for multilevel models of incidence is available upon request.
