# Supplementary material for: Geographic determinants of colorectal cancer in Louisiana
Source: Cancer Causes Control. 2022 Jan 7;33(4):525–32. doi: 10.1007/s10552-021-01546-7 (PMC8904347; doi:10.1007/s10552-021-01546-7)
Supplement: Supplementary file 1 — Supplementary file1 (DOCX 14 kb) [file 10552_2021_1546_MOESM1_ESM.docx]

**Supplemental Table 1.** Relative risk (RR) estimates and 95% confidence intervals from sensitivity analyses of colorectal cancer incidence, Louisiana 2008-2017.

|  | Urban - Rural | |  | Metro - Non-metro | |
| --- | --- | --- | --- | --- | --- |
|  | RR (95% CI) ^1^ | RR (95% CI) ^2^ |  | RR (95% CI) ^1^ | RR (95% CI) ^2^ |
| White Females |  | | White Females |  | |
| Rural | **1.09 (1.02,1.16)** | 1.05 (0.98,1.12) | Non-metro | **1.13 (1.08,1.20)** | **1.09 (1.03,1.15)** |
| Low SES | **1.14 (1.06,1.23)** | **1.11 (1.03,1.20)** | Low SES | **1.14 (1.06,1.23)** | **1.10 (1.01,1.18)** |
| Acadian | **1.16 (1.09,1.23)** | **1.15 (1.08,1.22)** | Acadian | **1.16 (1.09,1.23)** | **1.13 (1.06,1.20)** |
| White Males |  | | White Males |  | |
| Rural | **1.11 (1.04,1.18)** | 1.06 (1.00,1.13) | Non-metro | **1.16 (1.10,1.22)** | **1.11 (1.06,1.17)** |
| Low SES | **1.16 (1.08,1.24)** | **1.12 (1.04,1.21)** | Low SES | **1.16 (1.08,1.24)** | **1.11 (1.03,1.19)** |
| Acadian | **1.18 (1.11,1.25)** | **1.17 (1.10,1.24)** | Acadian | **1.18 (1.11,1.25)** | **1.14 (1.08,1.21)** |
| Black Females |  | | Black Females |  | |
| Rural | 1.02 (0.93,1.12) | 0.98 (0.89,1.07) | Non-metro | 1.03 (0.96,1.11) | 0.99 (0.92,1.07) |
| Low SES | **1.13 (1.05,1.21)** | **1.14 (1.07,1.23)** | Low SES | **1.13 (1.05,1.21)** | **1.14 (1.06,1.22)** |
| Acadian | **1.21 (1.09,1.33)** | **1.22 (1.11,1.35)** | Acadian | **1.21 (1.09,1.33)** | **1.23 (1.11,1.36)** |
| Black Males |  | | Black Males |  | |
| Rural | 1.00 (0.91,1.10) | 0.97 (0.88,1.06) | Non-metro | 1.03 (0.96,1.11) | 0.99 (0.92,1.07) |
| Low SES | **1.08 (1.01,1.16)** | **1.10 (1.02,1.18)** | Low SES | **1.08 (1.01,1.16)** | **1.09 (1.02,1.17)** |
| Acadian | **1.21 (1.10,1.33)** | **1.22 (1.11,1.35)** | Acadian | **1.21 (1.10,1.33)** | **1.23 (1.11,1.35)** |

Abbreviations: RR=relative risk, CI=confidence interval, SES=socioeconomic status.

^1^Adjusted for age (5 year groups).

^2^Adjusted for age (5 year groups) and other risk factors in the table (rural, low SES, Acadian).

Estimates that were statistically significant, at alpha=0.05, are shown in bold.
